# Effects of peripheral nerve injury on parvalbumin expression in adult rat dorsal root ganglion neurons

**DOI:** 10.1186/s12868-015-0232-9

**Published:** 2015-12-16

**Authors:** Tom Medici, Peter J. Shortland

**Affiliations:** School of Science and Health, Western Sydney University, Narellen Road, Campbelltown, NSW 2560 Australia; Centre for Neuroscience and Trauma, Blizard Institute, Barts and The London School of Medicine and Dentistry, Queen Mary University of London, Newark Street, London, E1 2AT UK; Queens Hospital, Romford, Essex, RM7 0AG UK

**Keywords:** Plasticity, Axotomy, Spinal nerve ligation, Dorsal root ganglion, Proprioceptor

## Abstract

**Background:**

Parvalbumin (PV) is a calcium binding protein that identifies a subpopulation of proprioceptive dorsal root ganglion (DRG) neurons. Calcitonin gene-related peptide (CGRP) is also expressed in a high proportion of muscle afferents but its relationship to PV is unclear. Little is known of the phenotypic responses of muscle afferents to nerve injury. Sciatic nerve axotomy or L5 spinal nerve ligation and section (SNL) lesions were used to explore these issues in adult rats using immunocytochemistry.

**Results:**

In naive animals, the mean PV expression was 25 % of L4 or L5 dorsal root ganglion (DRG) neurons, and this was unchanged 2 weeks after sciatic nerve axotomy. Colocalization studies with the injury marker activating transcription factor 3 (ATF3) showed that approximately 24 % of PV neurons expressed ATF3 after sciatic nerve axotomy suggesting that PV may show a phenotypic switch from injured to uninjured neurons. This possibility was further assessed using the spinal nerve ligation (SNL) injury model where injured and uninjured neurons are located in different DRGs. Two weeks after L5 SNL there was no change in total PV staining and essentially all L5 PV neurons expressed ATF3. Additionally, there was no increase in PV-ir in the adjacent uninjured L4 DRG cells. Co-labelling of DRG neurons revealed that less than 2 % of PV neurons normally expressed CGRP and no colocalization was seen after injury.

**Conclusion:**

These experiments clearly show that axotomy does not produce down regulation of PV protein in the DRG. Moreover, this lack of change is not due to a phenotypic switch in PV immunoreactive (ir) neurons, or de novo expression of PV-ir in uninjured neurons after nerve injury. These results further illustrate differences that occur when muscle afferents are injured as compared to cutaneous afferents.

## Background

Peripheral nerve injury disconnects sensory and motor axons from their peripheral targets and results in the production of regeneration associated genes such as α-tubulin, GAP43, CAP23, ATF3, and STAT3 that are important in the growth and functional recovery of damaged sensory and motor axons [1, 2]. Primary sensory neurons also show considerable plasticity when subjected to peripheral nerve injury. For example, directly injured neurons can upregulate neurotransmitters such as NPY, BDNF, galanin whilst adjacent uninjured neurons can increase their content of neurotransmitters such as substance P, CGRP, BDNF, galanin and ion channels such as TRPRV1 and P2X3 [3–5]. This plasticity is thought to contribute to the generation and maintenance of neuropathic pain [3, 6, 7]. However, the mechanisms that contribute to chronic pain syndromes are incompletely understood. Several different models have been developed to explore the contributions of primary afferents to chronic pain syndromes [3, 8, 9]. Most models employ injury to the sciatic nerve, a mixed peripheral nerve, or its branches. Interestingly, one study demonstrates the importance of muscle afferents to the pathobiology of nerve injury pain. When the gastrocnemius (muscle) or tibial (mixed) or sural (cutaneous) nerve was sectioned, mechanical and thermal hypersensitivity only occurred in nerve injuries involving muscle afferents [10].

The phenotypic responses of muscle afferents to nerve injury have been relatively little studied. In the DRG, muscle afferents are generally identified by their size, neurotrophic factor dependence, or their expression of particular neurotransmitters or proteins [11–18]. Retrograde tracing experiments show that muscle afferents are both myelinated and unmyelinated [16]. Muscle nociceptors are generally small and contain high levels of the neuropeptide CGRP [15, 16] whereas muscle spindle afferents are generally large-sized, myelinated fibres that express carbonic anhydrase, or the calcium binding proteins calretinin, calbindin, neurocalcin or parvalbumin [19–25]. Some of these markers, however, are not specific to muscle afferents, as they are also found in skin afferents [26–28]. Whilst parvalbumin is generally regarded as a reliable marker for proprioceptive afferents [22, 23], genetic studies have revealed that, by itself, it is not a selective marker of muscle proprioceptors nor a marker of muscle afferent nociceptors [26, 29].

Relatively few studies have assessed the effects of nerve injury on parvalbumin expression. Most of the published literature concerns injuries to branches of the trigeminal [30, 31] or sciatic [22, 32, 33] nerves and the general consensus appears to be that injury has little effect on parvalbumin expression in the affected DRGs. One possible reason for this is that a phenotypic change in expression in injured versus uninjured afferents occurs, such that parvalbumin expression is decreased in injured neurons and increased in uninjured neurons. As the sciatic nerve contains axons originating in the L4-6 DRGs, afferents from these ganglia are only partially affected by peripheral nerve injury. By assessing changes only in parvalbumin expression with this model it is not possible to tell whether a phenotypic change has occurred. Phenotypic switching in primary afferents is known to occur in nerve injury models [3, 5, 34]. This issue can be explored by using the regeneration associated transcription factor ATF3 [35]. ATF3 is not expressed in the DRG nuclei of uninjured neurons but is rapidly induced in all injured neurons following peripheral axotomy or spinal nerve injury [35]. By quantitatively assessing the number of co-labelled ATF3 and parvalbumin neurons after sciatic nerve injury, it is possible to determine the presence or absence of a phenotypic switch. A second, and more clear-cut, method involves using a spinal nerve ligation and section injury [36]. This essentially injures all axons in the L5 DRG but leaves axons in the adjacent L4 DRG largely unaffected [5, 34, 35]. Changes can then be compared by assessing the numbers of PV cells in the two adjacent ganglia.

Therefore, the aim of this study is to test the hypothesis that peripheral nerve injury induces a phenotypic switch in subpopulations of PV-ir neurons in the dorsal root ganglia.

## Results

### Naive animals

**Fig. 1:**
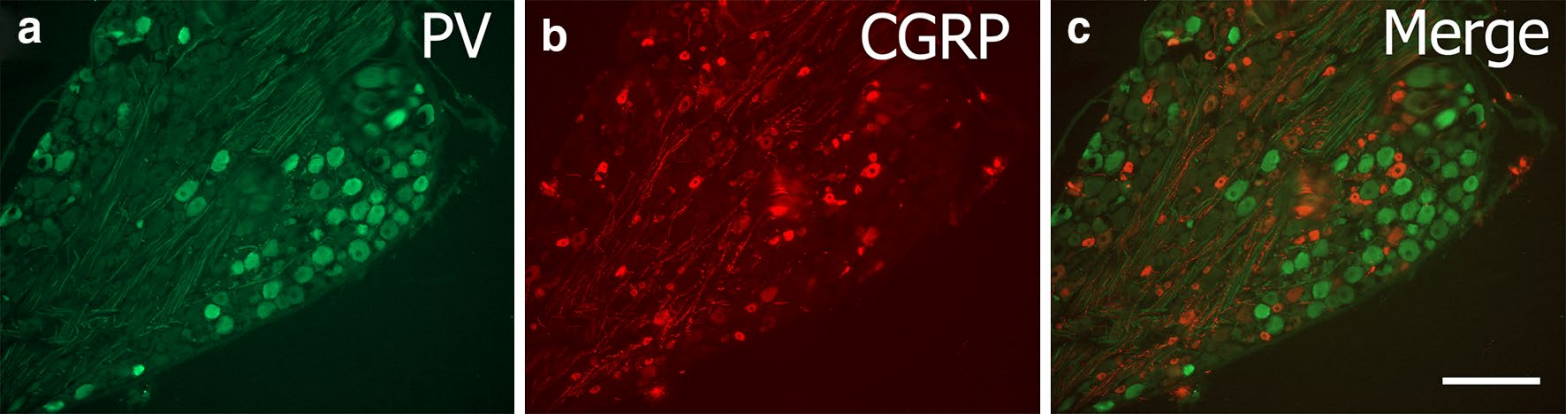
An example of parvalbumin and CGRP staining in naïve L5 DRG. **a** shows PV-ir (*green*), **b** shows CGRP-ir (*red*) staining and **c** is the merged image showing essentially no overlap between these populations. *Scale bar* 100 μm

PV-ir was expressed primarily in medium to large-sized DRG neurons (Fig. 1a). Quantification of expression in the L4 and L5 ganglia is shown in Table 1. Similar percentages were expressed in both ganglia and there was no statistical difference between the ganglia (*t*_7_ = 0.255, P > 0.5, 2-tailed test).

**Table 1:** Percentage expression of parvalbumin in L4 and L5 DRGs in naïve and sciatic axotomy groups

	L4 DRG	L5 DRG
Naïve (N = 6)	25.7 % ± 1.8	25.1 % ± 1.8
2 week sciatic nerve axotomy (N = 5)	23.1 % ± 1.9	21.7 % ± 1.5

CGRP-ir was predominantly expressed in small-sized DRG neurons with some expression in medium- and large-sized DRGs (Fig. 1b). Quantification of CGRP-ir expression in the naive L5 ganglia was 48.3 ± 1.9 % (N = 6). Colocalisation between PV-ir and CGRP-ir within the L5 DRG was negligible (1.9 ± 1.9 %) and, when present, was restricted to the large-sized neurons.

### Sciatic nerve axotomy

The expression of PV-ir in the L4 and L5 ganglia 2 weeks after sciatic nerve axotomy is detailed in Table 1. The percentage expression in these ganglia was not significantly different from naïve ganglia (L5 DRG: *F*_2,11_ = 1.800, P > 0.1, L4 DRG: *t*_7_ = 0.766, P > 0.5, 2-tailed test). The PV-ir in axotomised neurons was observed in the medium to large-sized cells of the ganglia, identical to that seen in naive ganglia.

**Fig. 2:**
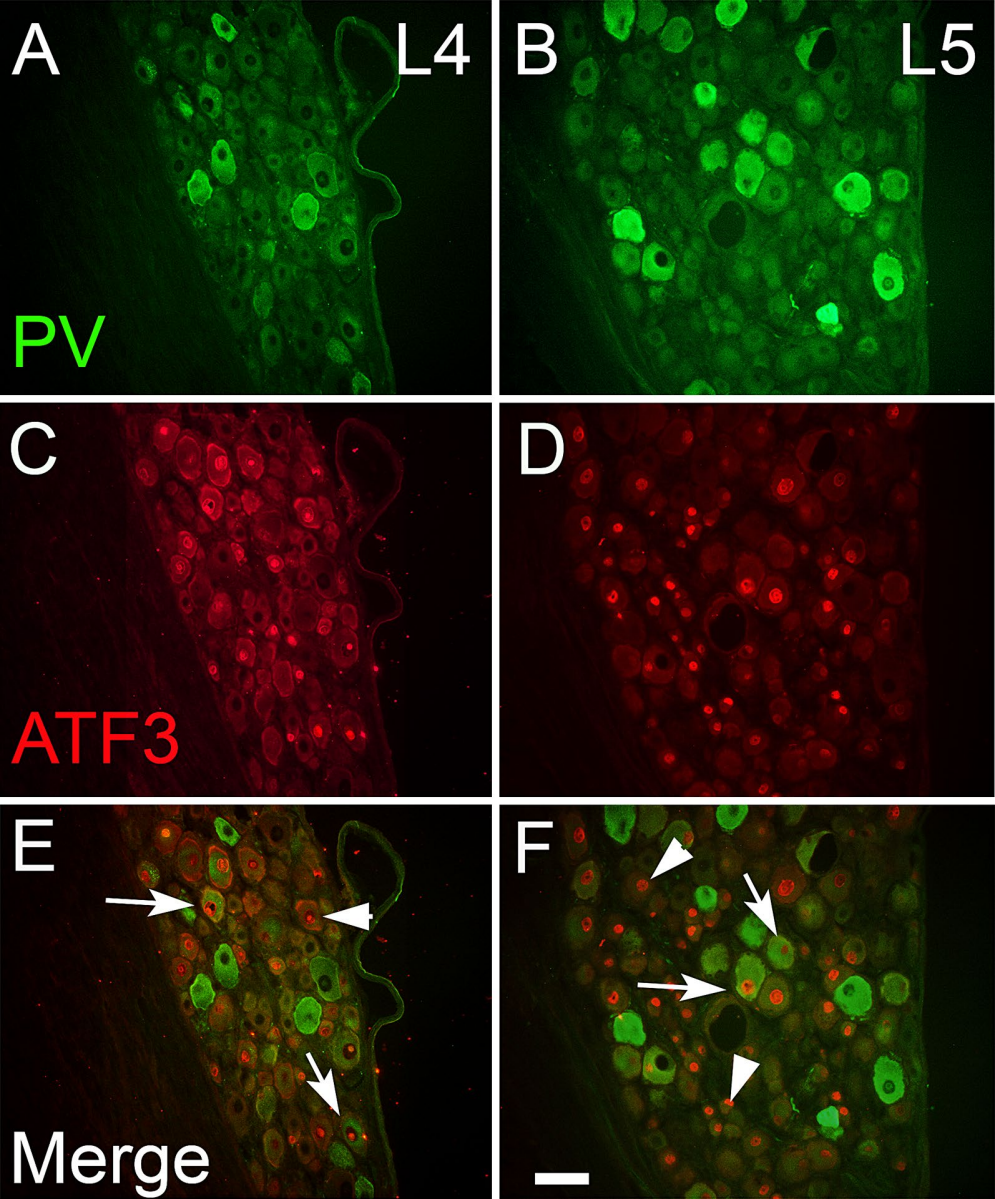
Parvalbumin (*green*) and ATF3 (*red*) staining in the ipsilateral L4 (**A**, **C**, **E**) and L5 (**B**, **D**, **F**) ganglia of rats with sciatic nerve axotomy 2 weeks previously. *Arrows* indicate double-labelled cells, *whilst arrowheads* denote cells only expressing ATF3. Both injured and uninjured cells express PV-ir. *Scale bar* 75 μm

It is possible that the unchanged percentage of PV-ir after sciatic nerve axotomy reflects a phenotypic switch in neurons such that injured neurons lose immunoreactivity and uninjured ones start to express PV de novo. To explore this possibility colocalisation with ATF3 was performed (Fig. 2). ATF3-ir was expressed in 50.7 % ± 4.9 of ipsilateral L5 DRG and 43.4 % ± 7.0 of the ipsilateral L4 ganglion cells, and these values were was not significantly different from each other (p = 0.21).

If a phenotypic switch occurs, one might expect to see little or no colocalisation with ATF3 after injury. However, this was not the case (Fig. 2). Analysis of double labelled sections showed that 23.7 % ± 3.7 of PV-ir cells (range 16–30 %) expressed ATF3 in the injured ipsilateral L4-5 ganglia. By itself, this result is insufficient to determine if a phentotypic switch is occurring.

### Spinal nerve ligation

To better explore the possibility of a phenotypic switch after injury, the experiment was repeated using the L5 spinal nerve injury model, which essentially damages all of the axons of the L5 ganglion neurons whilst leaving most of the axons of the adjacent L4 DRG intact [5, 34].

**Fig. 3:**
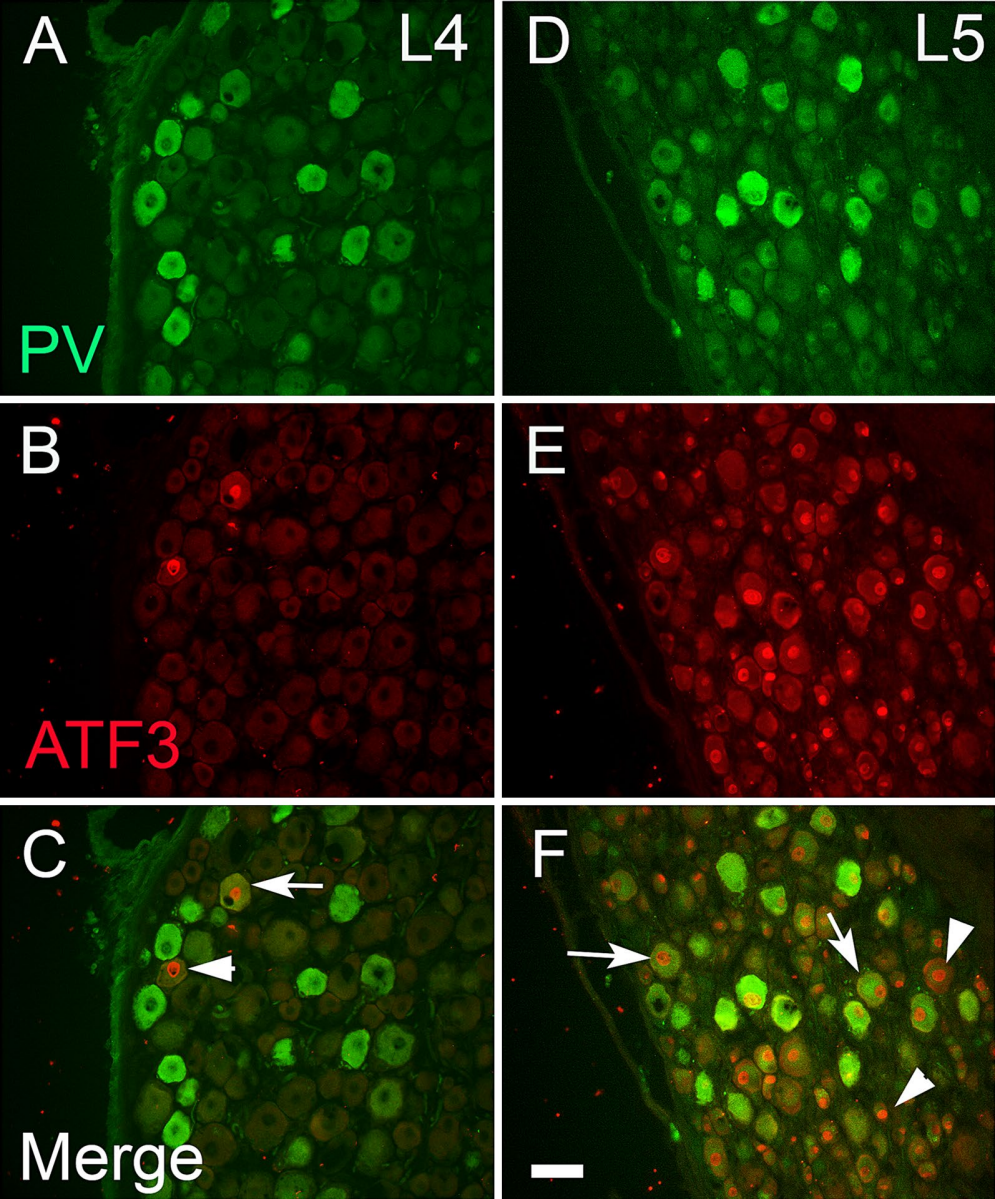
Parvalbumin (*green*) and ATF3 (*red*) staining in the ipsilateral L4 (**A**, **C**, **E**) and L5 (**B**, **D**, **F**) ganglia 2 weeks after L5 spinal nerve ligation and section. *Arrows* denote examples of double-labelled cells; *arrowheads* denote examples of cells that only express ATF3. Almost all PV-ir cells were ATF3-ir in the L5 ganglion (*arrows*). Very few ATF3 cells were seen in L4 DRG. *Scale bar* 75 μm

The L4 and L5 ganglia from both ipsilateral and contralateral sides to the L5 SNL were stained (Fig. 3) and quantified for immunoreactivity to PV, ATF3, and CGRP (Table 2). The mean percentage expression of PV-ir in the ipsilateral injured L5 ganglia was not significantly different from naive animals (*F*_1,8_ = 0.266, P > 0.5) or the contralateral side (*F*_1,8_ = 2.126, P > 0.1) or the ipsilateral L4 DRG (*F*_1,8_ = 0.103, P > 0.5).

**Table 2:** Effects of L5 spinal nerve ligation on percentage expression of different neurochemical parameters in the L4-5 DRG neurons 2 weeks after injury

(N = 5)	L4 DRG ipsilateral	L5 DRG ipsilateral	L5 DRG contralateral
Parvalbumin	23.0 % ± 1.2	22.8 % ± 0.9	22.2 % ± 0.8
CGRP	44.5 % ± 1.7	0	48.3 % ± 2.7
ATF3	2.8 % ± 0.9	92.8 % ± 0.9	0.3 % ± 0.1

ATF3-ir in the contralateral L5 DRG was negligible (Table 2). In the injured L5 ganglia, ATF3-ir was found in almost all cells (Fig. 3), confirming the completeness of the lesion. Essentially, nearly all PV-ir neurons coexpressed ATF3 (Table 2). In the ipsilateral L4 ganglia the average ATF3-ir was low (Table 2) but significantly more than the contralateral side (*F*_1,6_ = 7.242, P = 0.036), with the occasional PV-IR cell showing ATF3 staining (Fig. 3).

**Fig. 4:**
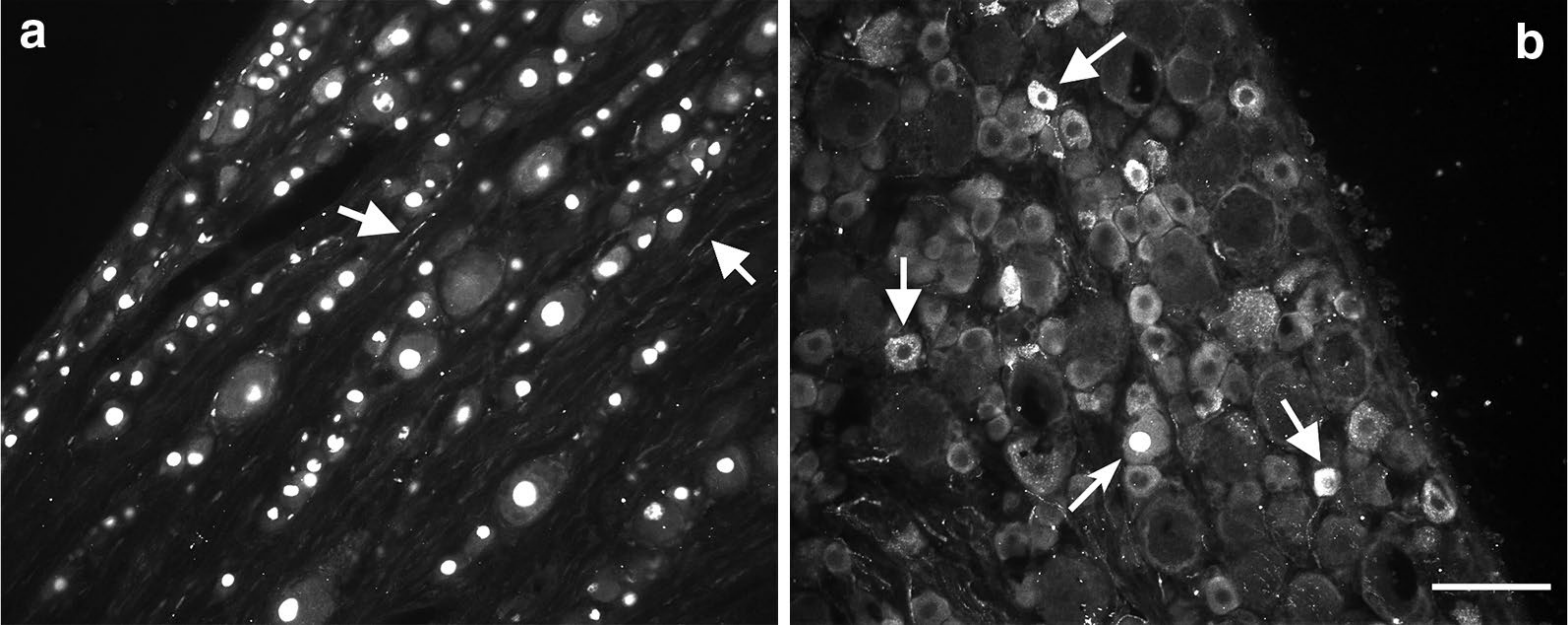
An example of ATF3 and CGRP staining in L5 DRGs 2 weeks after spinal nerve section. **a** L5i ganglion showing no CGRPr in ATF3 positive cells. CGRP axons were visible in the tissue (*arrows*) but the characteristic punctate staining in the cell cytoplasm is absent. **b** Contralateral ganglion showing many CGRP-ir cells (*arrows*). The *curved arrow* shows an example of an ATF3+/CGRP− cell. *Scale bar* = 50 μm

CGRP staining was essentially absent from the L5 DRG neurons in the injured ganglion, although some axonal staining could be detected (Fig. 4). The characteristic punctate staining in the cell cytoplasm that is normally present (Fig. 4b) is absent in the ipsilateral L5 DRG (Fig. 4a). In the ipsilateral L4 ganglia the average CGRP-ir was not significantly different from naïve animals (Table 2; p > 0.5) and colocalisation of CGRP in PV-ir cells was 0.6 ± 0.3 %.

## Discussion

PV is often used as a marker of a subpopulation of (muscle) proprioceptive afferents, comprising 10–33 % of cells in spinal and trigeminal ganglia [11, 19, 22, 23, 33, 37, 38]. This study found that 25 % of naïve rat L4-5 DRGs were PV-ir, consistent with these earlier reports. By itself, PV is not an exclusive marker of muscle proprioceptive afferents, as it is also expressed in Pacinian corpuscles, Merkel cells and lanceolate endings of cutaneous afferents [26]. Thus, since retrograde tracing from muscles was not used in this study we cannot be sure that all PV-ir cells in this study are muscle proprioceptors. However, only around 1.5 % of L4-5 PV-ir DRG cells arise from skin afferents [22] and a recent anatomical study showed that 27 % of lumbar dorsal root fibres were positive for α3 Na+/K+ ATPase, a specific marker of muscle afferents [13]. The similarity in percentage expression of PV-ir and α3 Na+/K+ ATPase implies that PV-ir afferents should express this ATPase transporter, although this awaits confirmation. Together, these suggest that the vast majority of PV-ir cells are muscle proprioceptors.

Whilst PV is almost exclusively expressed in large diameter muscle afferents [22], CGRP is expressed in both large and small diameter muscle afferents [16]. The large-sized neurons correspond to the group I-II muscle spindle and Golgi tendon organ (proprioceptive) afferents, whereas group III-IV afferents are small diameter muscle afferents associated with muscle metabolic responses, blood vessel innervation, sensations involved during exercise and muscle pain [39]. Colocalisation studies suggest that PV is found in DRGs that may also contain calbindin, carbonic anhydrase (CA) and cytochrome oxidase [11, 20]. This is consistent with the proposed role of PV in facilitation of fast, repeated, afferent firing of fast twitch muscles [40, 41]. CGRP is also expressed in large-sized DRG cells but the colocalisation of PV and CGRP was minimal in the present study, confirming earlier studies [23, 42].

### Plasticity of primary afferents after injury

DRG cells show considerable plasticity in response to peripheral nerve injury. Following injury, peptides such as CGRP and substance P are often down regulated in injured DRGs whilst other peptides such as galanin and neuropeptide Y are upregulated (reviewed by [4, 6]). This de novo expression also occurs in adjacent uninjured DRGs [3].Only a few studies have assessed the effects of axotomy on PV afferents and these have largely been in the trigeminal system [31, 43, 44]. The trigeminal system is different from the spinal system in that PV-ir is expressed in both the trigeminal ganglion (Vg), which contains cell bodies of cutaneous afferents and the mesencephalic nucleus (Vmes), which contains cell bodies of muscle afferents. When the inferior alveolar nerve was sectioned no change in PV numbers occurred in the Vg [31]. When the masseter nerve was sectioned there was a significant drop in PV-ir cells in Vmes between 4 and 14 days post injury [43]. However, as there was no baseline control values for PV-ir in Vmes, there is no certainty as to whether there was an overall decrease in expression by day 14 or whether there was an increase which normalised by day 14. Alternatively, since PV is expressed in periodontal ligament afferents in the Vg [44], the difference between the two studies may reflect a difference in the response to injury of cutaneous mechanoreceptors versus muscle afferents.

In spinal DRGs, previous injury studies suggest that PV numbers either did not change [22] or were transiently decreased [32]. These studies were at early time-points when changes in neurochemical phenotype are only beginning to start [45]. Additionally, these previous studies did not consider the possibility of a phenotypic switch between injured and uninjured neuronal populations [3, 34]. This study examined changes at only 1 time point, 2 weeks after injury. Whilst it is possible that changes occurred before then, previous studies have shown that peak changes in DRG neurotransmitter staining occur at 2 weeks post injury [4, 46]. Moreover, the percentage expression of ATF3 positive injured neurons remains unchanged over the first month after both types of nerve injury [5, 47]. Therefore, analysing co-labelling at this time point should maximise the changes of observing any differences as a result of injury. By using the SNL model and co-labelling ATF3 with PV, this study unequivocally showed that injury did not produce a phenotypic switch in PV staining from injured to uninjured neurons, and that PV-ir in DRGs is not regulated by nerve injury. This contrasts with small diameter neurons, whose phenotype changes with nerve injury due to the loss of target derived trophic factors like NGF and BDNF [46].

These results are also consistent with the lack of change of other markers in large-sized DRG neurons after injury: NF200, a marker of large myelinated afferents, is unchanged [45, 46] and α3 Na+/K+ ATPase is reported not to change in spinal nerve injury up to 1 week post injury [17]. Neuronal p75 levels in muscle afferents are unchanged after injury [48] as is TRPV2 expression [49]; TRPV2 has been shown to be colocalised to some muscle afferents [50]. CA, which is also localised to muscle proprioceptive afferents, also shows no change in expression after sciatic nerve injury [51]. Lastly, the lectin soybean agglutinin (SBA) is unchanged after injury for at least 3 months in the DRG [52]. SBA is expressed in about 50 % of small- and medium-sized muscle DRG cells [18] and is extensively co-expressed with CGRP but not CA [53]. It is possible that PV levels may decline later on, in a fashion similar to SBA and CA, but further studies are required to verify this; however, we see no change in PV expression at 4 weeks after sciatic injury (Shortland and Medici, unpublished observations).

Does this mean that large-sized, presumed, muscle proprioceptive afferents are incapable of phenotypic plasticity? Evidence suggests that at least some medium-large diameter muscle afferents upregulate NPY and galanin and BDNF [10, 54, 55]. Indeed, Zhou et al. [10] demonstrated that BDNF was increased in axotomised muscle but not skin afferents and this was critical to produce neuropathic pain. Most recently, Fukuoka et al. [56] have demonstrated an increase in Nav1.7 in axotomised putative proprioceptors in DRGs and the gracile nucleus following spinal nerve ligation. Taken together, these studies suggest that different neurotransmitters and receptors may be differentially regulated within single cells. CGRP and substance P have also been reported to increase in large sized DRG neurons [57, 58]. Whilst mRNA levels have confirmed this, protein expression studies have been less convincing. The majority of studies examining SP or CGRP-IR changes in the axotomised DRG have used immunofluorescence cytochemistry [5, 45, 59] and failed to confirm the earlier reports based on mRNA or DAB histochemistry, suggesting differential sensitivity based on methodology. In this study, CGRP-ir was almost completely depleted in the L5 DRG somas after spinal nerve injury, and even if a few large cells increase CGRP protein expression these results suggest it is not in PV-ir afferents.

### Potential roles for parvalbumin after nerve injury

PV is a calcium binding protein responsible for controlling the levels of intracellular calcium ions [60]. Calcium is vital for cell function and its concentration must be strictly regulated: too little and the cell cannot perform functions such as neurotransmitter release; too much and cellular mechanisms of apoptosis are triggered [61]. Support for PV’s ability to increase cell survival comes from animal studies of ALS pathogenesis. PV is only expressed in subsets of motoneurons such as Onuf’s nucleus and the oculomotor nuclei [62–65], areas often spared from cell death in ALS patients. When PV was genetically overexpressed in motoneurons in vitro, they were better able to withstand concentrations of extracellular calcium associated with excitotoxic cell death and showed an increased ability to survive compared to their wild-type counterparts [66].

Peripheral nerve injury also causes cell death as a result of loss of trophic support [67, 68] that is differentially distributed with respect to peripheral target, with a preference for loss of cutaneous versus muscle afferents [48, 69–71]. PV-ir cells are known to be trkC positive [12, 26] and levels of NT3 in peripheral tissues are higher at birth then they are in the adult. It is possible that PV-ir cells maintain their trophic support from the CNS where levels are higher [72]. Supporting this, neonatal nerve crush depletes PV-ir cells from the DRGs [73]. Given that adult axotomy does not deplete neuronal p75 and that BDNF is upregulated in trkC neurons [48, 55], it is probable that PV-ir cells are trophically supported by BDNF released from BDNF baskets that surround trkC cells containing p75 in an autocrine manner [51]. In contrast, injured small sized, cutaneous DRGs, lose p75 neuronal staining and do not upregulate BDNF after injury [10, 48] and so may be more vulnerable to death.

Nerve injury also generates spontaneous and ectopic activity that is associated with axotomised and intact muscle, but not skin, afferents [39, 74, 75] that starts within hours, lasts many weeks and is predominantly associated with A-fibres. As PV-ir is associated with fast twitch muscles it is tempting to speculate that it is these fibres that may be spontaneously active in the DRG. However, electrophysiological data suggests that the very large-sized DRG cells are not spontaneously active [75]. As more than 80 % of axotomised muscle afferents express BDNF after injury [10] it is likely that many of the PV-ir afferents will express BDNF. Since BDNF is a potent neuromodulator of neuronal activity [76, 77], it is BDNF that is the major contributor to pain states from muscle afferents [10] whereas PV may be important for the survival of neurons after injury. An additional, or alternate, possibility is that spontaneous discharges may affect sensory processing through up-regulation of Nav1.7 in axotomised muscle afferents at the level of the gracile nucleus [56].

## Conclusions

These results show that in two different models of peripheral nerve injury, the percentage of PV in DRG cells remains unchanged compared to naive or contralateral DRGs. Furthermore, by using ATF3 as a marker of injured neurons and by using the spinal nerve ligation and section model [5, 34], they unequivocally demonstrate, for the first time, that this is not the result of a phenotypic switch in expression from injured to uninjured cells, as numbers of PV-ir cells in the intact L4 DRG and the injured L5 DG remain unaltered. Therefore, PV positive afferents do not show the same phenotypic plasticity that is characteristic of other neuronal subpopulations such as nociceptors [4, 46]. These results further highlight the differential responses of muscle as compared to cutaneous afferents after peripheral nerve injury.

## Methods

All experimental procedures were carried out in accordance with the UK Scientific Procedures Act (1986) and guidelines set out by the International Association for the Study of Pain guidelines for the care and use of animals [78].

### Surgery

Sixteen adult Wistar rats (250–350 g) were used and split into 3 groups: naive (n = 6), sciatic nerve axotomy (n = 5) and L5 spinal nerve ligation and section (n = 5). All surgical procedures were carried out on the left side under sterile conditions as described in detail previously [3, 43]. Briefly, the sciatic nerve was exposed at mid-thigh level, ligated with a 4-0 nylon suture and cut distal to the suture. For the spinal nerve injury, the L5 spinal nerve was exposed, ligated with a 4-0 nylon suture and the nerve cut distal to it. Following nerve section, the wound was closed and topical antibiotics applied to the wound site to prevent post-operative infection. All animals survived post-operatively for 2 weeks before perfusion.

Rats were terminally anaesthetised and perfused with saline followed by 4 % paraformaldehyde (dissolved in 0.1 M phosphate buffer, pH7.4) fixative. The L4 and L5 DRGs were removed bilaterally and post-fixed for 2 h before being cryoprotected overnight in 30 % sucrose dissolved in phosphate buffered saline (PBS) solution. Ganglia were embedded in OCT (VMR International) and frozen prior to sectioning for immunohistochemical staining.

### Immunocytochemistry

Longitudinal sections (8–10 µm) were cut on a cryostat and thaw-mounted onto Superfrost plus (VWR International) slides. Each adjacent section was separated by 50–100 µm. Sections were blocked in 10 % normal donkey serum (Chemicon) for an hour and then incubated with goat anti-parvalbumin (1:8000, Swant, PVG214 RRID:AB_10000345) for 24 h at room temperature. Slides were then washed 3 times in PBS before incubation with fluorescein isothiocyanate (FITC)-conjugated donkey anti-goat IgG (Jackson ImmunoResearch, 1:400) for 1 h.

In double labelling studies, sections were first incubated in PV for 24 h, washed in PBS and then incubated with rabbit anti-ATF3 (1:200, Santa Cruz SC-188 RRID:AB_2258513), or rabbit anti-CGRP (1:2000, Affinity Research Products Ltd., UK) for 24–48 h at room temperature. Following three PBS washes, sections were then incubated in donkey anti-rabbit Cy3 (1:800, Jackson ImmunoResearch, USA) for 2 h at room temperature. In some slides, sections were also counterstained with 100 µg/ml DAPI (4,6-diamino-2-phenylindole, Sigma, UK) to reveal neuronal nuclei. All slides were cover-slipped with 8:1 glycerol:PBS solution. Controls for double labelling included reversing the order of the antibodies or omission of the primary or secondary antibodies. The characteristics and validity of these antibodies have been described previously [5, 46, 79].

Slides were examined on a Leica fluorescence microscope. For each animal, and each DRG, a minimum of 500 neuronal profiles (cells with clearly visible nuclei, confirmed by DAPI staining) were counted from 3 to 5 randomly chosen sections and the immunoreactivity (ir) was expressed as a percentage of all cells counted. PV-ir cells were considered positive if the intensity of staining was clearly above background staining seen in small-sized neurons in the same section.

In double labelled sections, the percentage of PV-ir cells expressing either ATF3-ir or CGRP-ir was assessed by switching between the DAPI, FITC and TRITC filter blocks. Photographs were taken with a Hamamatsu digital C4742-95 camera and plates assembled using Adobe Photoshop v5. The quantification was performed in a non-blinded fashion, since levels of ATF3-IR would give clues to which group was being quantified.

### Statistical analysis

All data is expressed as mean ± standard error of the mean. One-way ANOVA tests or independent samples t tests were carried out (using SPSS 16 statistical package, SPSS Inc., Chicago, IL, USA) to assess whether there was a statistically significant difference between groups; p < 0.05 was considered significantly different.

## Abbreviations

ir: immunoreactivity
ATF3: activating transcription factor 3
PV: parvalbumin
CGRP: calcitonin gene related peptide
FITC: fluorescein isothiocyanate
Cy3: cyanine dye 3
TRITC: tetrarhodamine isothiocyanate
DAPI: 4,6-diamino-2-phenylindole
DRG: dorsal root ganglion
IgG: immunoglobulin
